# P300 inhibition enhances gemcitabine-induced apoptosis of pancreatic cancer

**DOI:** 10.18632/oncotarget.10117

**Published:** 2016-06-17

**Authors:** Hiroaki Ono, Marc D. Basson, Hiromichi Ito

**Affiliations:** ^1^ Department of Surgery, Michigan State University, College of Human Medicine, Lansing, MI, USA; ^2^ Departments of Surgery, Basic Science and Pathology, University of North Dakota, School of Medicine and Health Sciences, Grand Forks, ND, USA

**Keywords:** p300, gemcitabine, histone acetyl transferase, DNA damage repair, pancreatic cancer

## Abstract

The transcriptional cofactor p300 has histone acetyltransferase activity (HAT) and has been reported to participate in chromatin remodeling and DNA repair. We hypothesized that targeting p300 can enhance the cytotoxicity of gemcitabine, which induces pancreatic cancer cell apoptosis by damaging DNA. Expression of p300 was confirmed in pancreatic cancer cell lines and human pancreatic adenocarcinoma tissues by western blotting and immunohistochemistry. When pancreatic cancer cells were treated with gemcitabine, p300 was recruited to chromatin within 24 hours, indicating the role in response to DNA damage. When p300 was gene-silenced with siRNA, histone acetylation was substantially reduced and pancreatic cancer cells were sensitized to gemcitabine. The selective p300 HAT inhibitor C646 similarly decreased histone acetylation, increased gemcitabine-induced apoptosis and thus enhanced the cytotoxicity of gemcitabine on pancreatic cancer cells. These findings indicate that p300 contributes to chemo-resistance of pancreatic cancer against gemcitabine and suggest that p300 and its HAT activity may be a potential therapeutic target to improve outcomes in patients with pancreatic cancer.

## INTRODUCTION

Although overall cancer mortality has declined over the past 2 decades in the United States, the mortality of patients with pancreatic cancer has not improved. [1] The poor efficacy of current chemotherapy for pancreatic cancer is a critical factor in these poor outcomes. Gemcitabine (2′, 2′-difluoro-2′-deoxycytidine, dFdC) is the current gold standard chemotherapeutic agent for the treatment of pancreatic cancer [2], but inherited or acquired drug resistance limits its cytotoxic efficacy. Therefore, a novel target to enhance current chemotherapy is clearly needed to improve the outcomes of patients with pancreatic cancer.

p300 is a 300 kDa global transcriptional coactivator with histone acetyltransferase (HAT) activity. p300 binds to chromosomal histones and modulates the chromatin conformation by acetylation of its histones, leading to activation of various gene transcriptions [3, 4]. Thus, p300 participates in diverse cellular functions including proliferation, differentiation and apoptosis [3]. In particular, p300 plays a critical role in DNA repair synthesis after DNA damage induced by ultraviolet or radiation [5, 6]. While p300 is considered to serve as a tumor suppressor gene that prevents carcinogenesis in normal epithelial cells [7, 8], p300 has often been observed to be overexpressed in various types of cancer and p300 overexpression in such cancers has been associated with worse patient outcomes [9–16]. The mechanisms by which p300 overexpression worsens cancer outcomes are unknown.

We hypothesized that p300 is associated with resistance to DNA-damaging chemotherapy and that targeting p300 can enhance the cytotoxicity of such chemotherapeutic agents in pancreatic cancer. We evaluated the effect on gemcitabine-induced DNA damage and apoptosis in pancreatic cancer of gene silencing of p300 and of pharmacological inhibition of p300 by the small molecule inhibitor C646. Our findings identify p300 as a novel molecular target to improve the efficacy of current chemotherapeutic regimens for patients with pancreatic cancer and improve their long-term outcomes.

## RESULTS

### p300 is aberrantly expressed in pancreatic cancer cells

**Figure 1:**
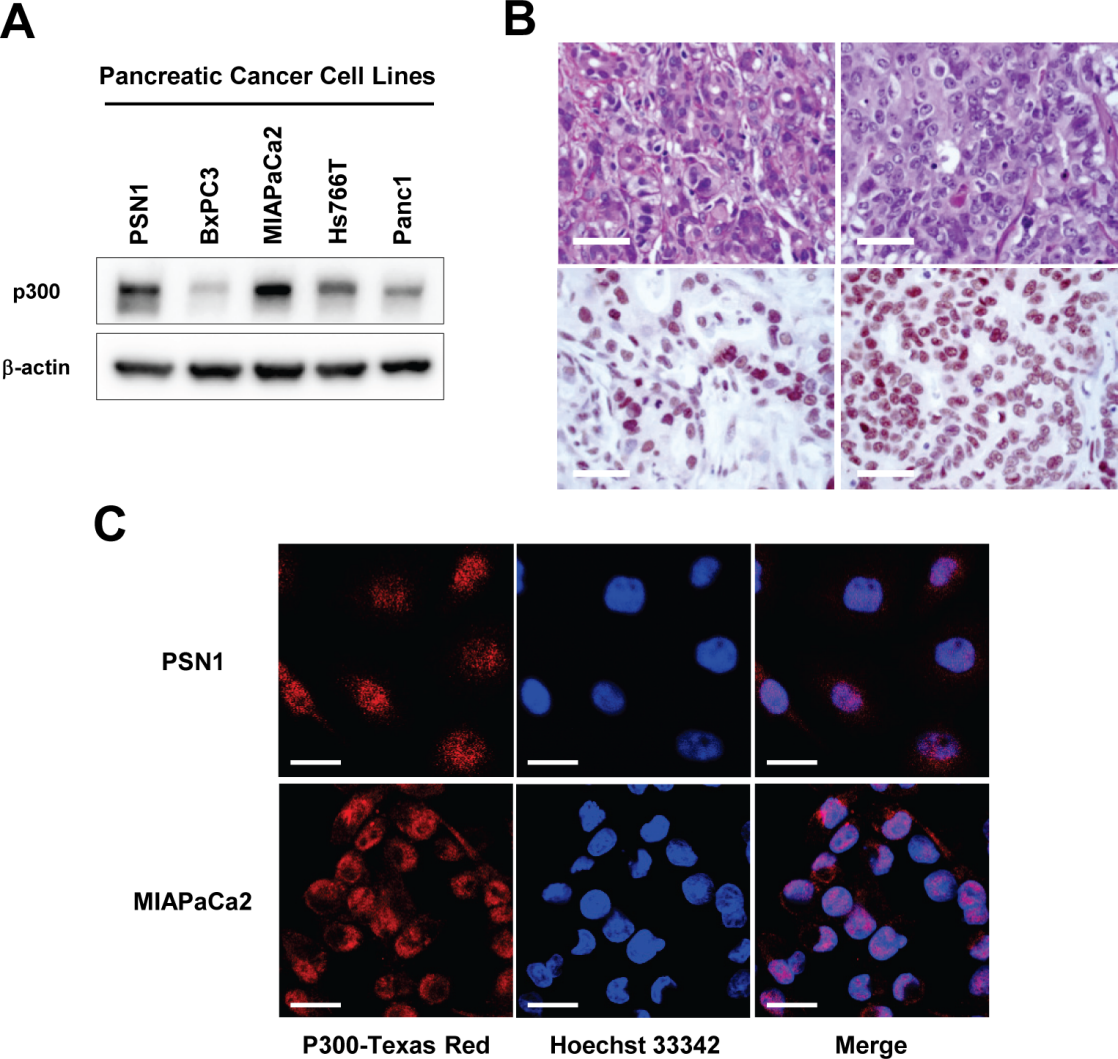
(**A**) Endogenous p300 expression in human pancreatic cancer cell lines. p300 expression levels were examined in 5 pancreatic cancer cell lines, PSN1, BXPC3, MIAPaCa2, Hs766T, and Panc1, by western-blotting. (**B**) Representative images for immunohistochemical stains for ductal adenocarcinomas with positive p300 expression. Upper panel: Hematoxylin-Eosin stain, Lower panel: Immunohistochemical staining with p300 antibody, Bars, 40 um. (**C**) Representative images for immunofluorescence staining of p300 expression in PSN1 and MIAPaCa2 cells. P300 was primarily localized in the nucleus in both cell lines. Bars, 20 um.

Several reports have demonstrated p300 overexpression in other cancers, including skin, liver, esophageal, nasopharyngeal, lung, breast, prostate and colorectal cancers, and have described the poorer prognoses of patients with tumors with higher p300 expression level than of patients with tumors that express less p300 [9–16]. However, there is no data regarding p300 expression in pancreatic cancer. Therefore, we first evaluated p300 expression in pancreatic cancer using five established human pancreatic cancer cell lines (PSN1, BXPC3, MIAPaCa2, Hs766T, and Panc1) and eleven pancreatic ductal adenocarcinoma tissue samples from patients who had undergone pancreatic resection. In the panel of pancreatic cancer cell lines, endogenous p300 expression at various levels was detected in all cancer cells by western blotting (Figure 1A). Immunohistochemical staining of surgically resected pancreatic ductal adenocarcinoma also demonstrated p300 expression (Figure 1B). Among the eleven tumors we studied, nine tumors (81%) showed strong staining by the p300 antibody, primarily in the nucleus, while only two (19%) tumors showed undetectable or minimal immunoreactivity.

We further analyzed the localization of p300 in cancer cells by immunofluorescent staining. As shown in Figure 1C, p300 immunoreactivity localized to the nucleus of pancreatic cancer cells.

### Chromatin-bound p300 is increased by gemcitabine exposure

**Figure 2:**
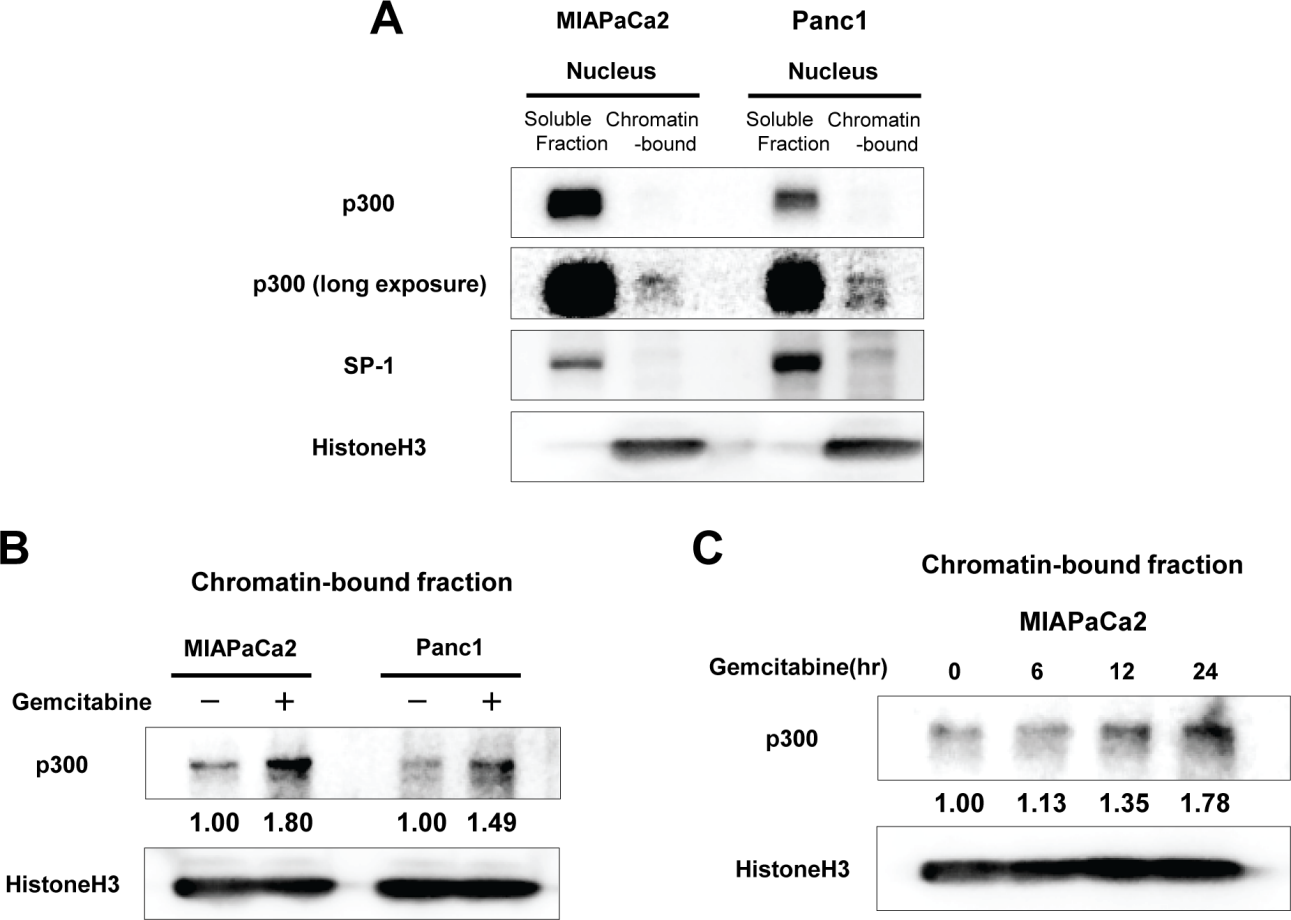
(**A**) Distribution of p300 in the nucleus of MIAPaCa2 and Panc1 cells: Isolated nuclear fractions were separated into soluble and chromatin-bound fractions and resolved by western blotting. Most p300 was present as a soluble form in the nucleus of pancreatic cancer cells while much less p300 was bound to chromatin in the baseline condition. SP-1 and Histone H3 were used as positive control for soluble nuclear proteins and for chromatin-bound proteins, respectively. (**B**) Effects of gemcitabine treatment on chromatin-bound p300 in MIAPaCa2 and Panc1 cells. The cells were treated with gemcitabine (at 50 nM for MIAPaCa2 and 10 uM for Panc1) for 24 hours and then the nuclear proteins were isolated. The doses of gemcitabine for each cell line were selected where the cytotoxic effect of gemcitabine reached to the plateau. Gemcitabine exposure induced p300 recruitment to chromatin at 24 hours in pancreatic cancer cells. The numbers at the bottom indicate the relative intensity of the bands for chromatin-bound p300 to corresponding controls (normalized by HistoneH3). (**C**) The levels of chromatin-bound p300 in MIAPaCa2 during gemcitabine treatment (50 nM) for 24 hours. Chromatin-bound p300 increased over the course of gemcitabine exposure within 24 hours.

We next investigated the dynamics of expression of p300 in pancreatic cancer cells upon exposure to DNA damage induced by gemcitabine. Because p300 in the nucleus has been shown to exist as either a soluble form or a chromatin-bound form [17], we separated the nuclear protein into a soluble fraction and a chromatin-bound fraction. Although the chromatin-bound form of p300 represented a much smaller proportion of the entire nuclear p300 pool, the chromatin-bound p300 is likely to be important in the DNA damage response (Figure 2A). When the cells were exposed to gemcitabine, the chromatin-bound form of p300 was increased within 12–24 hours in MIAPaCa2 and Panc1 cells (Figure 2B, 2C), while the soluble form of p300 remained unchanged (Supplementary Figure S1).

### Gene silencing of p300 increased gemcitabine-induced apoptosis in pancreatic cancer

**Figure 3:**
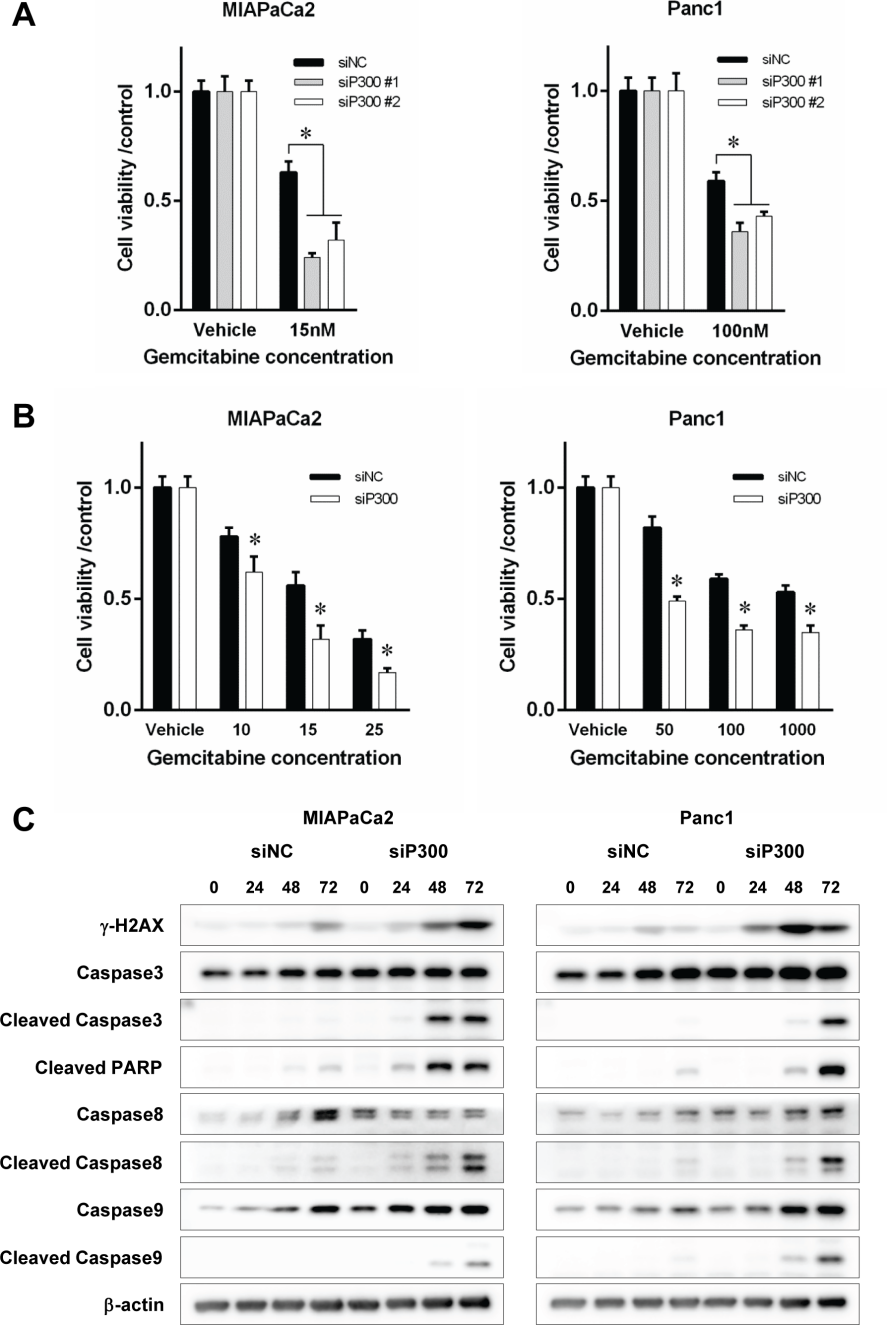
(**A**) Prior to gemcitabine treatment, cells (0.5–1.0 × 10^3^) were treated with siP300 or siNC for 48 hours. Then, cells were treated with gemcitabine for 96 hours. Cell viability was assessed by WST-8 assay at 96 hours and was normalized to controls (*p < 0.05 vs controls treated with non-specific siRNA). (**B**) Cell viability after gemcitabine treatment at various doses for 96 hours with siP300 or siNC. Cells were sensitized by p300 gene-silencing (*p < 0.05 vs siNC control, respectively, by ANOVA with a post hoc Bonferroni correction). (**C**) Effects of p300 gene-silencing on gemcitabine-induced DNA damage and apoptosis. Degree of DNA damage was evaluated by γ-H2AX and apoptosis by cleaved Caspase-3, 8, 9, and PARP. Cells were pretreated with siP300 or siNC for 48 hours prior and were treated with gemcitabine at 15 nM for MIPaCa2 and 100 nM for PANC1, respectively. Gene-silencing of p300 increased gemcitabine-induced DNA damage and apoptosis at 72 hours.

**Figure 4:**
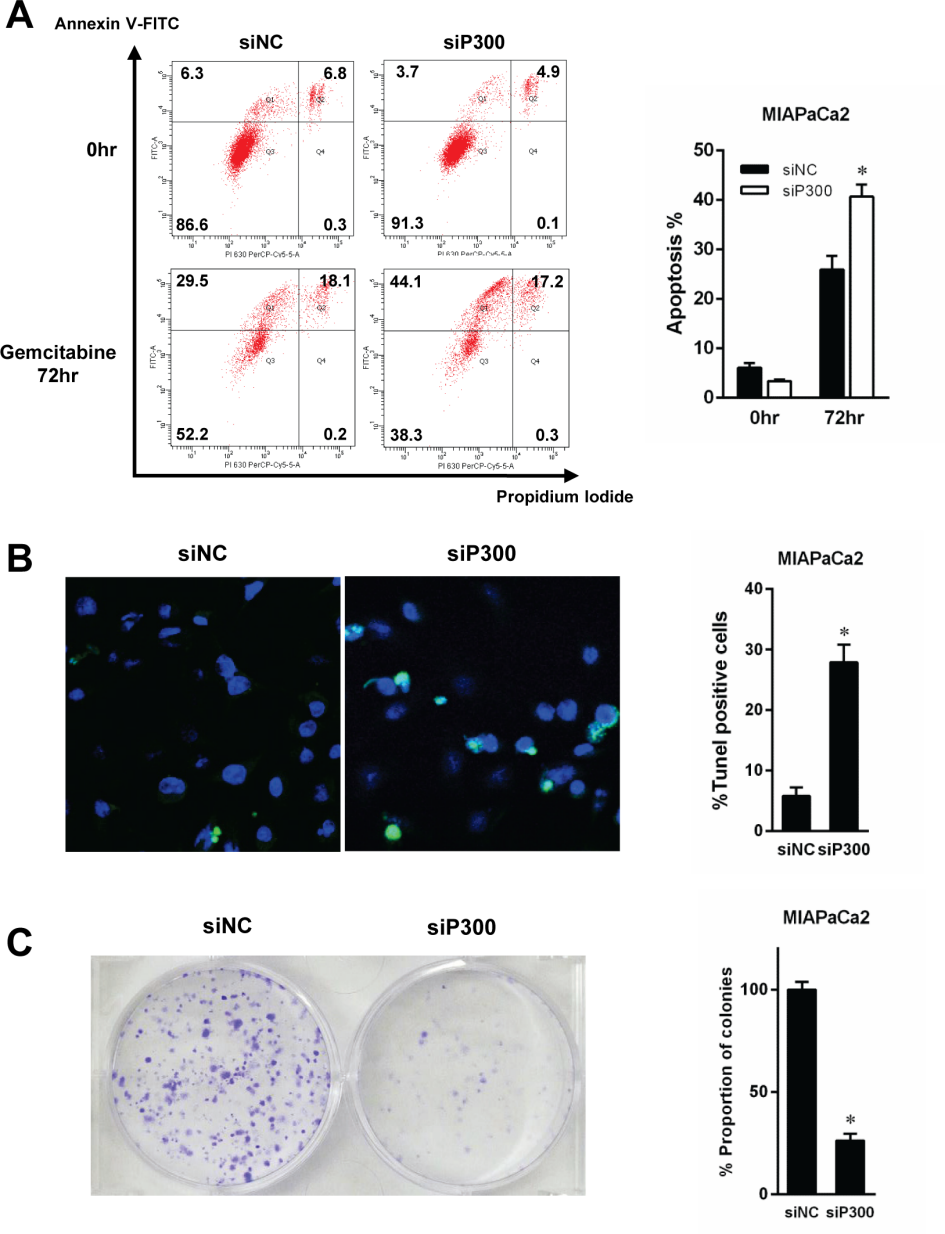
Gemcitabine-induced apoptosis was evaluated by flow cytometry (A) and TUNEL staining (B) (**A**) MIAPaCa2 cells were treated with gemcitabine at 15 nM for 72 hours and fixed with 70% ethanol at −20°C overnignt. Fixed cells were stained with for annexin V-FITC and propidium iodide (PI). The proportion of apoptotic cells was significantly increased by p300 gene silencing compared to the cells treated with non-specific siRNA (*p < 0.05). (**B**) MIAPaCa2 cells were treated with gemcitabine at 20 nM for 96 hours for TUNEL staining. TUNEL positive cells were stained with Alexa Fluor 488 according to manufacturer's instructions. The proportion of TUNEL-positive cells was determined by calculating the number of TUNEL-positive cells/the number of Hoechst 33342 staining cells per each field. The left panels depicted the representative photographs with tunnel stains. The number of Tunel positive cells was significantly greater in the cells treated with siP300 than the counterpart control treated with non-specific siRNA. (*p < 0.05). (**C**) Colony forming assay. MIAPaCa2 cells were treated with gemcitabine (20 nM) for 24 hours, then changed to fresh media without gemcitabine, and incubated for 9 days. P300 gene-silencing enhanced the long-term anti-tumoral effect of gemcitabine on MIAPaCa2 cells (*p < 0.05).

Based on the previously reported role of p300 in DNA damage response and our observation of recruitment of p300 to chromatin after gemcitabine exposure, we hypothesized that forced reduction of p300 in pancreatic cancer cells might sensitize the cells to gemcitabine. We gene-silenced p300 in pancreatic cancer cells using specific siRNA for 48 hours, and then exposed the cells to gemcitabine. The depletion of p300 protein at 48 hours caused by specific siRNA was confirmed by western-blotting as shown in Supplementary Figure S2. There was no difference in the efficacy of siRNA to suppress p300 expression between the different cell lines or siRNA obtained from the different venders. The number of viable cells after 96 hours of gemcitabine treatment was significantly decreased by p300 gene silencing compared to the control cells for each cell line (63% vs 24% and 32% for MIAPaCa2 siP300 #1 and #2 and 59% vs 36% and 43% for Panc1 siP300 #1 and #2, *p* < 0.05, respectively, Figure 3A). siP300#1 and #2 were different siRNA targeting p300 sequences from different vendors, so the similarity of effects between siP300#1 and siP300#2 makes the effects of reducing p300 expression that we observed unlikely to be non-specific effects. p300 gene silencing sensitized cancer cells to gemcitabine (*p* < 0.05, respectively) (Figure 3B). This reduction of viable cells with gemcitabine treatment was caused by increased gemcitabine-induced DNA damage and apoptosis. As shown in Figure 3C, p300 gene silencing increased γ-H2AX, a surrogate marker for double strand breaks in DNA, as well as markers for the apoptotic pathway including cleaved caspase 3, 8, 9 and PARP. Increased gemcitabine-induced apoptosis by p300 gene silencing was confirmed by other experiments using flow cytometry and Tunnel staining assay (Figure 4A, 4B). Furthermore, a colony forming assay showed the increased long-term anti-tumoral effect of gemcitabine by p300 gene silencing on pancreatic cancer cells (Figure 4C).

### Inhibition of p300 HAT activity by small molecule inhibitor C646 enhanced the cytotoxicity of gemcitabine against pancreatic cancer cells

**Figure 5:**
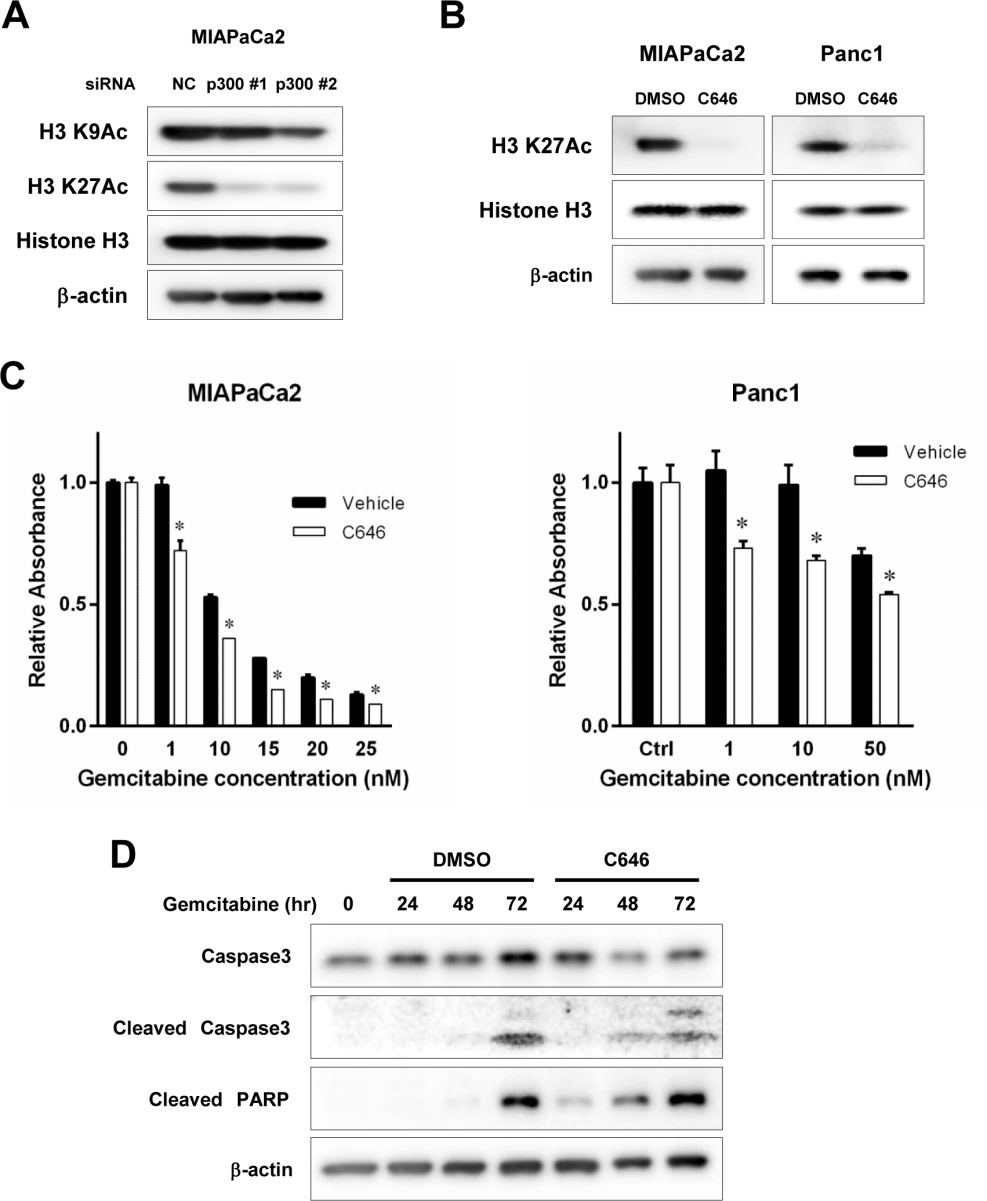
(**A**) Effects of p300 gene silencing on histone acetylation. P300 was gene-silenced using 2 different siRNAs targeting p300 and its effect on actetylation of H3K9 and H3K27 was assessed. Non-specific siRNA served as control. MIAPaCa2 cells were treated with siRNA for 72 hours. Acetylated H3K27 (H3K27Ac) was reduced by p300 gene silencing, while Acetylated H3K9 (H3K9Ac) was not affected. (**B**) The effect of C646 on p300 HAT activity. Cells were treated with C646 at 30 uM for MIAPaCa2 and 40 uM for Panc1 for 48 hours and then cell lysates were applied for western blotting to evaluate acetylated H3K27 as a surrogate of p300 HAT activity. C646 inhibited p300 HAT activity. (**C**) The effect of C646 on gemcitabine cytotoxicity. Cells were treated with gemcitabine alone or gemcitabine and C646 (30 uM for MIAPaCa2 and 40 uM for Panc1). The numbers of viable cells were normalized to those of the control cells without gemcitabine treatment. C646 sensitized the cells to gemcitabine (*p < 0.05 vs vehicle treated control, respectively, by two way ANOVA with a post hoc Bonferroni correction). (**D**) Effects of C646 on gemcitabine-induced apoptosis. MIPaCa2 cells were treated with gemcitabine at 20 nM and C646 at 30 uM through 72 hours. The expression of apoptotic markers were increased by C646 compared to the control cells treated with DMSO.

Finally, we tested the effects of p300 HAT inhibition on gemcitabine-induced apoptosis in pancreatic cancer using a small molecule inhibitor. C646 suppresses p300/CBP HAT activity and induces cell cycle arrest with growth suppression in other types of cancers [18, 19]. H3K27 (27^th^ lysine residue in Histone H3) has been reported as specific target of p300 HAT. [20] Indeed, when we gene silenced p300 with specific siRNA, acetylation of H3K27 was suppressed, while acetylation of other residue, for example, H3K9 was not (Figure 5A). Thus, we used the acetylation of H3K27 as a surrogate for p300 dependent HAT activity. When pancreatic cancer cells were treated with C646 at 30 uM for MIAPaCa2 and 40 uM for Panc1, the reductions in acetylated H3K27 were confirmed at 48 hours (Figure 5A). The doses of C646 for each cell were determined as the dose where the effect of C646 on H3K27 reached to the plateau. Of note, C646 was previously reported to inhibit H3 histone acetylation at within a range of 10 uM through 50 uM for other types of cancer cells. [13, 21, 22] HAT inhibition by C646 increased the cytotoxic effect of gemcitabine at 96 hours for pancreatic cancer cell lines (Figure 5B) and increased gemcitabine-induced apoptosis was confirmed by western blotting for cleaved Caspase 3 and PARP (Figure 5C). These findings suggest that p300 protects pancreatic cancer cells against gemcitabine-induced DNA damage, at least partially by HAT-dependent manner and targeting p300 HAT activity can enhance the therapeutic efficacy of gemcitabine against pancreatic cancer.

## DISCUSSION

Accumulating evidence has suggested an association between p300 overexpression and worse long-term survival of patients with various types of cancer, including lung, liver, colorectal, breast and esophageal cancer [9–16]. As p300 is involved in diverse biological activities both in benign epithelial and cancerous cells, the mechanism by which the overexpressed p300 impairs treatment outcomes of patients with cancer is not yet clearly defined. In the current study, we demonstrated dynamic changes in p300 expression in pancreatic cancer cells upon exposure to gemcitabine and suggested that p300 may contribute to pancreatic cancer chemo-resistance by protecting cells from gemcitabine-induced apoptosis.

An important role for p300 in DNA repair synthesis has been suggested previously in the cellular model with UV or ionizing radiation (IR) induced DNA breaks [5, 6]. Consistent with our observations in the present study, p300 was shown to be recruited at the sites of DNA break induced by UV or IR in human cells and facilitate DNA repair. Although p300 cannot promote DNA repair by itself, p300 has been considered to serve as a cofactor for multiple mechanisms of DNA repair synthesis [6, 17, 23, 24]. For instance, proliferating cell nuclear antigen (PCNA) is known to accumulate at the radiation-induced DNA damage sites and promote subsequent DNA repair. [25] Hasan and colleagues demonstrated that p300 forms a complex with PCNA which was essential in DNA replication to repair damaged DNA [5], and that p300 was necessary to recruit PCNA to the site of DNA damage. Ogiwara and colleagues also showed that p300 was crucial to the recruitment of other key molecules such as KU70 and KU80 for DNA repair and chromatin remodeling [6]. Gemcitabine is known to cause DNA double strand breaks by incorporated into DNA and induce apoptosis when cells fail to repair them, [26] and thus this p300-dependent DNA repair mechanism should be one of determinants for the cytotoxicity of gemcitabine against cancer cells.

Histone acetylation has been considered critical to many biological functions including DNA repair synthesis and chromatin remodeling at DNA damage sites [27]. Our study also indicated that p300-dependent histone acetylation plays an important role in DNA damage response after gemcitabine exposure in pancreatic cancer cells. Since the spectrum of target residues for p300 in histone proteins is wide and overlapped by the target residues by other proteins with HAT activity, it is not practically possible to identify the exact histones residue responsible for p300-mediated protection from gemcitabine-induced DNA damage. While the conformational change of the chromatin with acetylation of histones by p300 and the subsequent the recruitment of various DNA binding cofactors have been implicated as the primary mechanism of p300-mediated DNA repair [6, 17, 23, 24], Ogiwara and colleagues described other possible mechanisms by which p300 may also influence DNA repair. They reported p300-mediated transcriptional activation of DNA repair genes including BRCA1 and RAD51, upon IR induced DNA damage [23]. In our model of DNA damage by gemcitabine in pancreatic cancer, however, gemcitabine-induced DNA damage and apoptosis did not change by gene silencing of BRCA1 or RAD51 (data not shown), and thus the change in DNA repair gene expression upon the DNA damage stimuli may not contribute to the effect of p300 inhibitor on the cytotoxic efficacy of gemcitabine.

In summary, these results suggest that p300 may play an important role in protecting pancreatic cancer cells from apoptosis upon gemcitabine-induced DNA damage. Because p300 targeting by either siRNA or a small molecule p300 inhibitor enhances the cytotoxic efficacy of gemcitabine, p300 activity may therefore be a therapeutic target and further *in-vivo* experiments to test the therapeutic efficacy of p300 targeting are warranted.

## MATERIALS AND METHODS

### Materials

Culture media (DMEM), fetal bovine serum (FBS) and penicillin/streptomycin (P/S) were obtained from Gibco (Grand Island, NY). Anti-p300 (N-15; sc-584) and anti-SP1 (PEP2; sc-59) antibodies were obtained from Santa Cruz Biochemistry (Santa Cruz, CA). Anti-Caspase3 (8G10; #9662), anti-cleaved Caspase3 (5A1E; #9664), anti-Caspase8 (8G10; #9662), anti-Caspase9 (8G10; #9662), anti-cleaved PARP (D64E10; #5625), anti-γ-H2AX (20E3; #9718), anti-Acetyl Histone H3 (Lys9) (C5B11; #9649), anti-Acetyl Histone H3 (Lys27) (D5E4; #8173), and anti-Histone H3 (D1H2; #4499) antibodies were obtained from Cell Signaling Technology (Danvers, MA). Anti-β-actin antibody (AC-15; A5441) was obtained from Sigma-Aldrich (St. Louis, MO). Gemcitabine hydrochloride (G6423) and the p300 inhibitor C646 (SML0002) were obtained from Sigma-Aldrich.

### Cell cultures

The human pancreatic cancer BxPC3, MIAPaCa2, Panc1, and PSN1 cell lines were obtained on January 2012 from the American Type Culture Collection (ATCC, Manassas, VA). The Hs766T and PSN1 cell lines were obtained on April 2014. These cancer cell lines were authenticated by ATCC with DNA profiling using STR analysis before purchase. Cancer cells were maintained in DMEM medium containing 10% FBS and 1% P/S in a humidified 37°C, 5% CO_2_ chamber.

### Human tissues

Archived tissue slides were obtained from the Department of Pathology at Sparrow Hospital, Lansing MI. The use of archived specimens for this study was approved by the Michigan State University and Sparrow Hospital Institutional Review Boards. Tissue archives of pancreatic ductal adenocarcinoma from 11 different patients were included in this study. Heat-induced epitope retrieval was performed with citrate buffer (pH 6.0). Anti-p300 antibody was diluted in 1/100 with Normal Antibody Diluent (NAD) (Scytek – Logan, UT) and incubated for 1 hour at room temperature. Antigen-antibody reactions were visualized with the avidin-biotin-peroxidase complex system (R.T.U. Vectastain Elite ABC Reagent; Vector Laboratories, Burlingame, CA, USA).

### Western-blotting analysis

Western-blotting was performed as previously described. [28] Protein bands were visualized and their intensities were quantified using Odyssey Fc Imaging System (LI-COR Biosciences, Lincoln, NE). β-actin served as a loading control marker for normalization of each lane. All exposures for densitometry were within the linear range. Western-blotting was repeated at least three times with similar results and representative blots are presented.

### Gene silencing by small interfering RNA

Loss-of-function analysis was performed using siRNA targeting p300 (ON-TARGETplus SMARTpool #L-003486-00-0005, Dharmacon, Lafayette, CO). An alternative sequence of siRNA targeting p300 (Invitrogen, St Louis, MO: Stealth RNAi-P300HSS103259 sense 5′-GGAUUCGUCUGUGAUGGCUGUUUAA-3′, antisense- 5′-UUAAACAGCCAUCACAGACGAAUCC-3′) was also used with similar results. ON-TARGETplus Non-Targeting pool siRNA (#D-001810-10, Dharmacon) served as a negative control. Each siRNA (20 nmol/l) was transfected into pancreatic cancer cells using Lipofectamine RNAiMAX (Invitrogen) according to the manufacturer's instructions. The knockdown of each target gene was confirmed by western-blotting.

### Cell viability assay

Cell numbers were evaluated by an assay based on a colorimetric water-soluble tetrazolium salt, WST-8 (Cell Counting Kit-8; Dojindo Molecular Technologies, Gaithersburg, MD), as previously described. [29] In brief, 0.5–1.5 × 10^3^ cells per well were seeded in 96-well plates and incubated at 37°C. Following each treatment with or without gemcitabine, the absorbance of each well was measured at 450 nm using a μQuant Microplate Spectrophotometer (BioTek Instruments, Winooski, VT) and was within the linear range of the assay.

### Clonogenic survival assay

Long-term cell survival was evaluated by clonogenic survival assay as described. [30] Cells (1.0 × 10^3^ per well) were seeded in a 6 well plate in triplicate. After overnight incubation, attached cells were treated with gemcitabine for 24 hours. Culture medium was subsequently changed to fresh medium without gemcitabine. Cells were incubated for at least a week, and cells growing into colonies were stained with 0.3% crystal violet solution. A cluster of stained cells was considered as a colony when it included at least 50 cells.

### Apoptosis assay

Apoptotic cells were quantified by flow cytometry (LSRII; BD Bioscience, San Diego CA) following annexin V-FITC and propidium iodide (PI) staining using Annexin V-FITC Apoptosis Detection Kit (Sigma). DNA fragmentation was analyzed by TUNEL assay. Cells were fixed in 4% formaldehyde diluted in phosphate buffered saline for 15 minutes and permeabilized with 0.25% Triton™ X-100 in PBS for 20 minutes. TUNEL assay was performed with the Click-iT TUNEL Alexa Fluor 488 imaging kit according to the manufacturer's instructions. TUNEL-positive cells were counted from four randomly selected fields. The percent of TUNEL-positive cells was determined by calculating the ratio of the number of TUNEL-positive cells to the number of Hoechst 33342 staining cells in each field. This assay was performed in duplicate.

### Immunofluorescence staining

Cells were fixed in 4% formaldehyde diluted in phosphate buffered saline (PBS) for 15 minutes, permeabilized with 0.2% Triton™ X-100, and treated with blocking buffer (1% BSA in PBS), and then incubated overnight with the primary antibody at 4°C. Cells were then incubated with the Texas Red-conjugated secondary antibody (Texas Red^®^ goat anti-rabbit IgG, T2767, Life Technologies, Grand Island, NY) for 1 hour at room temperature.

### Fractionation of nuclear proteins

Whole cell lysates from cells were further fractionated into subcellular components using the Subcellular Protein Fractionation Kit for Cultured Cells (Pierce Biotechnology, Rockford, IL), according to manufacturer's instructions. The soluble nuclear extract and the chromatin-bound nuclear extract were used in this study. Following subnuclear fractionation, protein concentration was determined by BCA Protein Assay (Pierce Biotechnology). Five ug of protein from each extract was resolved by 10% SDS-PAGE and evaluated by western-blotting accordingly.

### Statistical analysis

Drawing figures, fitting curves, calculating IC50, and statistical analyses were performed with GraphPad Prism6 software (GraphPad Software Inc., San Diego, CA). Unless otherwise specified, experiments were conducted in triplicate in independent settings and the values presented represent their means, compared using Student's *t*-test for single comparison or ANOVA with a post hoc Bonferroni correction for multiple comparisons as appropriate. *P*-values less than 0.05 were considered significant.

## SUPPLEMENTARY MATERIALS FIGURES


