# Supplementary material for: P300 inhibition enhances gemcitabine-induced apoptosis of pancreatic cancer
Source: Oncotarget. 2016 Jun 17;7(32):51301–10. doi: 10.18632/oncotarget.10117 (PMC5239476; doi:10.18632/oncotarget.10117)
Supplement: Supplementary file 1 [file oncotarget-07-51301-s001.pdf]

P300 inhibition enhances gemcitabine-induced apoptosis of pancreatic cancer

Supplementary Materials

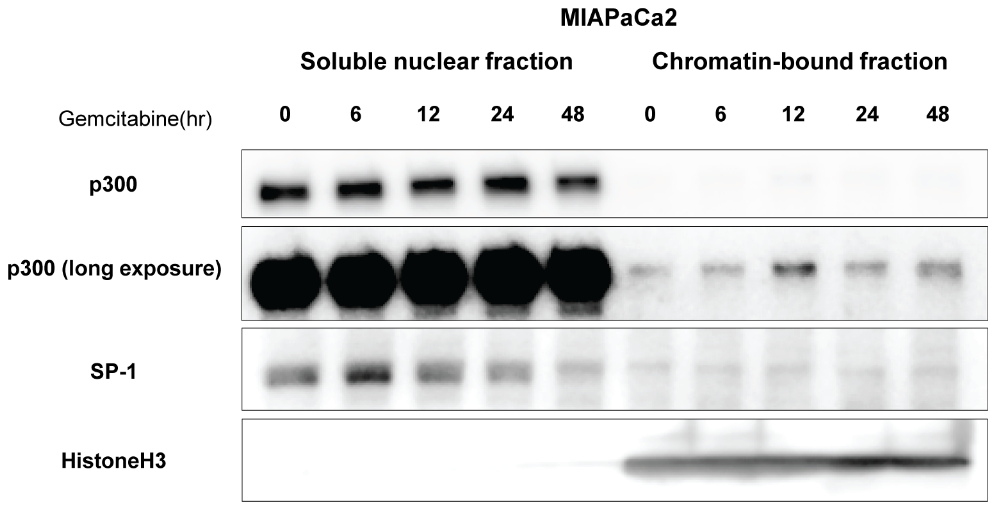

Supplementary Figure S1: p300 expression in soluble fraction and chromatin-bound fraction of MIAPaCa2 cells during gemcitabine treatment. Cells were treated with 25 nM gemcitabine. p300 expression in soluble fraction did not change within 48 hours, while p300 was recruited to chromatin, response to gemcitabine treatment.

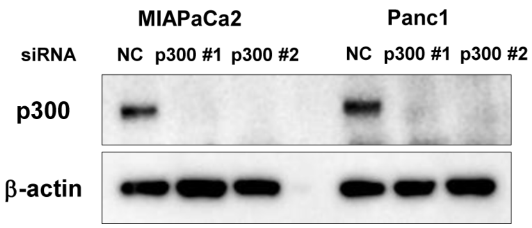

Supplementary Figure S2: The effects of p300-specific siRNA on p300 protein expression. p300 expression was suppressed by p300-specificsiRNAs in both MIAPaCa2 and Panc1 cells at 48 hours. The efficacy of siRNAs from different venders were the same.
